# CD44^+^ cytokeratin-positive tumor cells in blood and bone marrow are associated with poor prognosis of patients with gastric cancer

**DOI:** 10.1007/s10120-018-0858-2

**Published:** 2018-07-28

**Authors:** Antoni Szczepanik, Marek Sierzega, Grażyna Drabik, Anna Pituch-Noworolska, Piotr Kołodziejczyk, Marek Zembala

**Affiliations:** 1 First Department of Surgery, Jagiellonian University Medical College, 40 Kopernika Street, Kraków, 31-501 Poland; 2 Department of Clinical Immunology, Jagiellonian University Medical College, 265 Wielicka Street, 30-663 Kraków, Poland

**Keywords:** Gastric cancer, Circulating tumor cells, Disseminated tumor cells, CD44, Cytokeratins, Liquid biopsy

## Abstract

**Background:**

The phenotypic heterogeneity of circulating tumor cells (CTC) in peripheral blood and disseminated tumor cells (DTC) in bone marrow is an important constraint for clinical decision making. Here, we investigated the implications of two different subpopulations of these cells in gastric cancer (GC).

**Methods:**

GC patients (*n* = 228) who underwent elective gastric resections were prospectively examined for CTC/DTC. The cells obtained from peripheral blood and bone marrow aspirates were sorted by flow cytometry and CD45^−^ cells expressing cytokeratins (8, 18, and 19) and CD44 were identified by immunofluorescent double staining.

**Results:**

Ninety-three (41%) patients had cytokeratin-positive tumor cells in either blood or bone marrow, while cells expressing CD44 were found in 22 (10%) cases. CK^+^CD44^+^ cells were significantly more common among patients with distant metastases (50 vs 19%, *P* = 0.001), while no such correlations were demonstrated for CK^+^CD44^−^ cells. Detection of CK^+^CD44^+^ cells, but not CK^+^CD44^−^, was associated with significantly shortened survival. Moreover, the Cox proportional hazards model identified CK^+^CD44^+^ cells as a negative prognostic factor with an odds ratio of 2.38 (95% CI 1.28–4.41, *P* = 0.006).

**Conclusion:**

CD44^+^ phenotype of cytokeratin-positive cells in blood and bone marrow is an independent prognostic factor in patients with gastric cancer.

**Electronic supplementary material:**

The online version of this article (10.1007/s10120-018-0858-2) contains supplementary material, which is available to authorized users.

## Introduction

Gastric cancer (GC) is the third leading cause of cancer death worldwide, accounting for nearly 9% of fatalities related to human malignancies [1]. Except for some Asian countries with effective screening programs, most patients are diagnosed with advanced cancers and ultimately die from metastatic disease [2]. Therefore, much interest has been devoted to the understanding of the mechanisms associated with the initiation and progression of the metastatic cascade.

Circulating tumor cells (CTCs) in the peripheral blood and disseminated tumor cells (DTCs) in the bone marrow are a common phenomenon in patients with various malignancies, including gastric cancer [3, 4]. These two pools serve as a reservoir for cancer cells detached from the primary tumor prior to their inoculation to the premetastatic niche, and play a crucial role in the formation of distant metastases [5]. Detection of CTCs and DTCs offers many potential benefits for clinically relevant strategies aimed at early diagnosis and monitoring of anti-cancer treatment [6]. However, despite numerous efforts to better characterize both cell types for clinical decision making, many aspects remain controversial.

A recent meta-analysis of gastric cancer patients suggested that the presence of either CTCs or DTCs was significantly associated with impaired disease-free survival (HR 3.42, 95% CI 2.39–4.91) while CTCs markedly worsened only the overall survival (HR 2.13, 95% CI 1.13–4.03) [7]. However, a considerable degree of heterogeneity was observed between individual studies, related to differences in population characteristics as well as cell sampling and detection methods. Another important aspect of inconclusive results found in some studies could also been caused by the phenotypic heterogeneity of CTCs/DTCs, including cell clones of varying malignant potential [8–10].

The transmembrane glycoprotein CD44 was one of the first molecules identified on gastric cancer stem cells [11]. Subsequent studies demonstrated that CD44 in primary tumors was associated with a more advanced stage, larger tumor size, and more prevalent lymph node metastases [12]. Moreover, interactions between the glycoprotein and various adhesion molecules, like hyaluronic acid, were found to regulate stem cell migration and homing [13, 14]. Therefore, it seems reasonable to expect that CTCs/DTCs expressing CD44 are potentially more important for disease progression that those positive only for cytokeratins.

Our initial observations suggested that cytokeratin-positive CTCs may have only limited prognostic implications [15]. We have also demonstrated that the presence of CTCs positive for CD44 was associated with their presence in primary tumors, but only about 10% of cytokeratin-positive CTCs showed expression of CD44 [16, 17]. Taking into account the ambiguities related to circulating and disseminated tumor cells, the purpose of this study was to investigate the importance of two different phenotypic profiles of cancer cells in blood and bone marrow of patients with resectable gastric cancer subject to long-term follow-up.

## Materials and methods

### Patients and treatments

Patients with histologically proven adenocarcinoma of the stomach admitted between 2001 and 2007 were prospectively examined for the presence of tumor cells in blood and bone marrow. A total of 228 consecutive patients who underwent elective gastric resections were selected for this study, and the remaining 90 were excluded due to unresectable cancers. In general, patients with distant metastases identified by preoperative imaging were not qualified for surgery. However, if oligometastatic disease limited to the peritoneum or liver was found at laparotomy the decision about gastric resection was at the discretion of the operating surgeon.

The extent of surgery was classified as defined by the recent guidelines (Japanese Gastric Cancer, 2011). Tumor staging followed the seventh edition of the American Joint Committee on Cancer classification for gastric cancer [18]. All data were prospectively collected and recorded in a dedicated database. Patients were followed by clinical examinations performed every 3–6 months after discharge and dates of death were verified by the census registry office. The Bioethics Committee of the Jagiellonian University approved the protocol of this study and all patients gave informed consent before taking part in the study.

### Detection of circulating and disseminated tumor cells

Samples of peripheral blood (20 ml) and bone marrow aspirates (5 ml) from the iliac crest were taken before initiating any treatment. Pelleted cells were incubated with a lysing solution (Becton–Dickinson Biosciences, San Jose, CA) for 10 min, repeated 3–4 times to remove erythrocytes. The remaining cells were washed in phosphate-buffered saline (PBS) and adjusted to the concentration of 1 × 10^7^ cells/ml in PBS. Subsequently, the cells were stained with monoclonal mouse anti-human CD45 (phycoerythrin labeled) antibodies (DAKO, Glostrup, Denmark) and sorted into CD45^+^ and CD45^−^ populations using flow cytometry (FACS Vantage SE, BD Biosciences) equipped with the TurboSort (BD Biosciences) option and Aerosol Protection System (Flexoduct International ApS, Greve, Denmark). The Innova Enterprise II ion laser (Coherent, Santa Clara, CA, USA) operating at 488 nm was used as a light source. Sorting was performed using a 70-mm nozzle tip with a drop drive frequency of 65 kHz, 1.5 drop envelopes and a ‘normal’ sorting mode. Sorted CD45^−^ cells were collected into polystyrene Falcon 2057 tubes (BD Biosciences, Bedford, MA, USA) precoated with fetal calf serum and maintained in a refrigerated bath recirculator (Neslab Instruments, Portsmouth, NH, USA). About 1 × 10^6^ of CD45^−^ cells (1 × 10^6^ cells/ml) were used to prepare slides. The slides were dried, fixed with a mixture of ethanol and acetone (1:1 vol), and then stained for cytokeratins (CK). CK staining was carried out with PE-conjugated A45-B/B3 monoclonal antibodies (5 μg/ml) (Micromet GmbH, Germany), which recognize common epitopes of CK 8, 18 and 19. Subsequently, the slides positive for CK were double stained with CD44 monoclonal antibodies (G44-26, Becton–Dickinson, San Jose, CA) conjugated with fluorescein (FITC). The cells were identified by two independent investigators under a BX60 fluorescent microscope (Olympus, Tokyo, Japan), including morphological appearance of cancer cells, and documented with a DP10 camera (Olympus, Japan). At least 300 cells were examined per slide. The samples were regarded as positive when at least 3 cells CK^+^ per slide were found.

### Statistical analysis

All continuous variables are reported with their median and interquartile range (IQR) while categorical data are reported as proportions. Statistical significances of the differences in categorical and continuous variables were analyzed by Chi-square and Mann–Whitney *U* tests where appropriate. Survival data were analyzed according to the Kaplan–Meier method and the log-rank test was used to detect differences between groups. Multivariate analysis was performed using a Cox proportional hazards model with a backward stepwise selection procedure. The probability for entering the model was 0.05 and for removal from the model 0.100. All tests were two-sided and *P* < 0.050 was considered statistically significant. Statistical analysis was performed using the IBM^®^ SPSS^®^ Statistics 24 software package (IBM Corporation, NY).

## Results

### Patient characteristics

There were 151 males and 77 females with a median age of 63 years (Table 1). Circulating tumor cells (CTC) were found in the peripheral blood of 31 patients, while disseminated tumor cells (DTC) were identified in 106 cases. The median number of CK-positive cells in blood or bone marrow was 3 and the same median was found for CD44-positive cells in both compartments. Table 2 shows the prevalence of CTC and DTC in patients with various tumor stages.

**Table 1:** Patient demographics and clinicopathological parameters (*N* = 228)

Age (years) (median, IQR)	63 (53–70)
Male/female, *n* (%)	151 (66)/77 (34)
ECOG performance status, 0–1/2–3, *n* (%)	184 (81)/44 (19)
Tumor location, *n* (%)	
Upper third	42 (18)
Middle third	41 (18)
Distal third	121 (53)
Whole stomach	24 (11)
Primary tumor (T, AJCC 2010), *n* (%)	
T1	27 (12)
T2	36 (16)
T3	100 (44)
T4	65 (29)
Lymph node metastases (N, AJCC 2010), *n* (%)	
N0	55 (24)
N1	23 (10)
N2	24 (11)
N3a	53 (23)
N3b	73 (32)
Distant metastases (M, AJCC 2010), *n* (%)	
M0	177 (78)
M1	51 (22)
Tumor stage (AJCC 2010), *n* (%)	
I	38 (17)
II	60 (26)
III	79 (35)
IV	51 (22)
Type of surgery, *n* (%)	
Total gastrectomy	150 (66)
Distal gastrectomy	56 (25)
Proximal gastrectomy	22 (10)
Residual tumor, *n* (%)	
R0	130 (57)
R1/R2	98 (43)
Perioperative chemotherapy, *n* (%)	132 (58)
Circulating tumor cells, *n* (%)	
Negative	197 (86)
CK^+^CD44^−^	22 (10)
CK^+^CD44^+^	9 (4)
Disseminated tumor cells, *n* (%)	
Negative	122 (53)
CK^+^CD44^−^	91 (40)
CK^+^CD44^+^	15 (7)

*IQR* interquartile range, *ECOG* Eastern Cooperative Oncology Group, *AJCC* American Joint Committee on Cancer 2010

**Table 2:** Prevalence of circulating tumor cells and disseminated tumor cells according to tumor staging

Stage	Circulating tumor cells			Disseminated tumor cells		
	Negative (*n* = 197)	CK^+^CD44^−^ (*n* = 22)	CK^+^CD44^+^ (*n* = 9)	Negative (*n* = 122)	CK^+^CD44^−^ (*n* = 91)	CK^+^CD44^+^ (*n* = 15)
I	35 (18)	1 (5)	2 (22)	19 (16)	18 (20)	1 (7)
II	51 (26)	8 (36)	1 (11)	36 (30)	22 (24)	2 (13)
III	69 (35)	8 (36)	2 (22)	42 (34)	33 (36)	4 (27)
IV	42 (21)	5 (23)	4 (45)	25 (20)	18 (20)	8 (53)

Numbers in parentheses are percentages

### Correlation of the presence of CTCs/DTCs to clinicopathologic parameters

Table 3 summarizes correlations between the presence of CK- and CD44-positive cells in either blood or bone marrow and various clinicopathologic parameters. There were no significant differences between patients negative for CTC/DTC and those with CK-positive cells in either compartment. However, cells with CD44 on their surface were about twofold more common in patients with distant metastases (19 vs 50%, *P* = 0.001). Moreover, CD44-positive cells were more prevalent among poorly differentiated primary tumors (73 vs 50%, *P* = 0.055). When peripheral blood and bone marrow were analyzed separately, CD44-positive cells in the latter compartment (i.e., DTC) were associated with a twofold higher risk of distant metastases (Supplementary Tables 1 and 2). M1 disease was also more prevalent among cases with CD44-positive cells in blood (CTC), but the observed difference was not statistically significant, likely due to the small population of patients.

**Table 3:** Correlations of clinicopathological parameters with circulating and disseminated tumor cells

Parameter	CK/CD44 staining			*P*		
	Negative (*n* = 113)	CK^+^CD44^−^ (*n* = 93)	CK^+^CD44^+^ (*n* = 22)	Negative vs CK^+^CD44^−^	Negative vs CK^+^CD44^+^	CK^+^CD44^−^ vs CK^+^CD44^+^
Age (years) (median, IQR)	65 (55–70)	62 (53–70)	63 (52–69)	0.204^†^	0.652^†^	0.757^†^
Female (*n*, %)	39 (35)	32 (34)	6 (27)	0.987^*^	0.510^*^	0.522^*^
Tumor grade (*n*, %)				0.867^*^	0.055^*^	0.073^*^
Well or moderate Poor	56 (50) 57 (50)	45 (48) 48 (52)	6 (27) 16 (73)			
Primary tumor (*n*, %)				0.799^*^	0.648^*^	0.553^*^
T1–T2 T3–T4	31 (28) 82 (72)	27 (29) 66 (71)	5 (23) 17 (77)			
Metastatic lymph nodes (*n*, %)	83 (73)	70 (75)	20 (91)	0.766^*^	0.078^*^	0.110^*^
Distant metastases (*n*, %)	21 (19)	19 (20)	11 (50)	0.738^*^	0.001^*^	0.004^*^

*IQR* interquartile range, *CK* cytokeratin

*Chi-square test, ^†^Mann–Whitney *U* test

### Prognostic implications of CTCs/DTCs

**Fig. 1:**
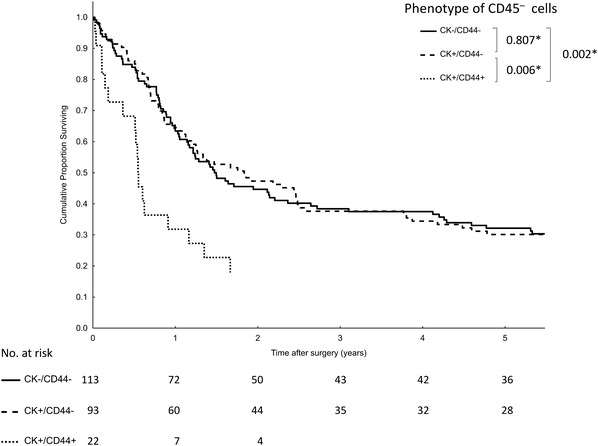
Kaplan–Meier analysis of overall survival according to the presence of cytokeratin (CK) and CD44-positive tumor cells in peripheral blood and bone marrow (*P, log-rank test)

**Fig. 2:**
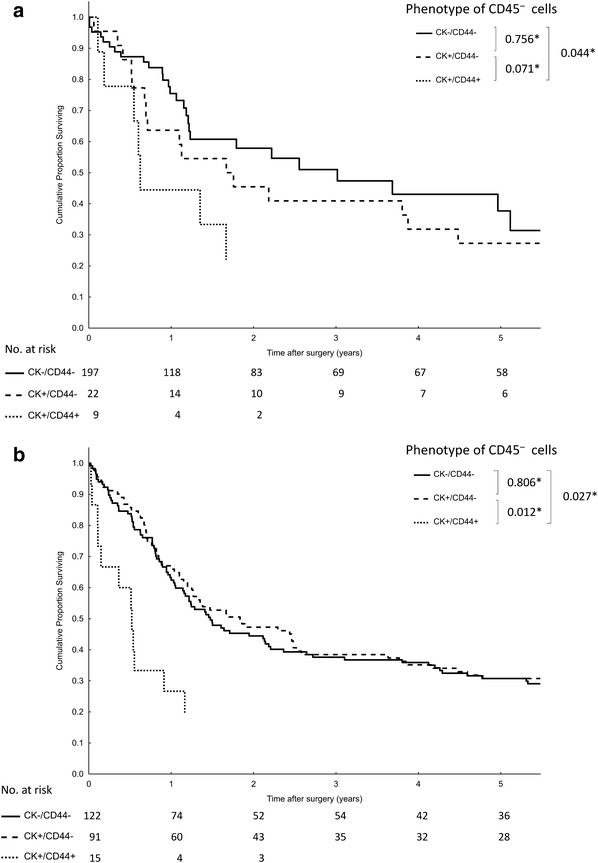
Kaplan–Meier analysis of overall survival according to the presence of cytokeratin (CK) and CD44-positive tumor cells (*P, log-rank test). **a** peripheral blood; **b** bone marrow

At the time of final follow-up (September 2016), 177 of 228 patients (78%) had died, and the median follow-up for surviving subjects was 99 months (range 76–119 months). The overall median survival was 17.3 months (95% CI 13.1–21.5). A univariate survival analysis with putative prognostic factors is illustrated in Table 4. Presence of CD44-positive cells in either blood or bone marrow significantly shortened median survival (6.7 months) compared to patients negative for CTC/DTC (22.3 months) and those with CK-positive cells (22.3 months) (Fig. 1). Moreover, this effect was still eminent if both compartments were analyzed separately (Fig. 2). However, CK-positive cells did not influence prognosis for DTC and CTC. A proportional hazards model constructed with only those variables that significantly affected survival in the univariate analysis identified five independent prognostic factors (Table 5), including CD44 positive cells with an odds ratio of 2.38 (95% CI 1.28–4.41; *P* = 0.001). Similar results were obtained in the population of patients subject to curative R0 resections (Supplementary Table 3).

**Table 4:** Univariate analysis of prognostic factors

Parameters	Category	Median survival months (95% CI)	Odds ratio (95% CI)	P*
Age (years)	< 65	20.3 (8.1–32.5)	1.00	0.081
	> 65	15.6 (10.8–20.4)	1.30 (0.96–1.75)	
Gender	male	17.1 (10.3–23.9)	1.00	0.292
	female	17.3 (11.5–23.1)	0.84 (0.61–1.16)	
ECOG performance status	0 or 1	25.9 (15.1–36.8)	1.00	0.043
	2 or 3	13.9 (11.1–16.7)	1.42 (1.01–1.83)	
Tumor location	whole stomach	8.5 (6.5–10.6)	1.00	0.007
	upper third	22.6 (12–33.2)	0.47 (0.27–0.83)	
	middle third	14.0 (9.9–18.1)	0.67 (0.39–1.15)	
	distal third	22.3 (11–33.6)	0.46 (0.29–0.75)	
Tumor grade	G1	32.2 (15.3–49.0)	1.00	0.011
	G2	23.0 (11.9–34.2)	1.01 (0.58–1.71)	
	G3	10.9 (8.3–13.4)	1.62 (0.97–2.69)	
Depth of infiltration (AJCC)	T1	147.6 (64.3–230.8)	1.00	< 0.001
	T2	90.5 (28.8–152.2)	1.28 (0.62–2.64)	
	T3	24.8 (18.2–32.4)	3.54 (1.92–6.51)	
	T4	12.8 (8.2–19.4)	8.31 (4.40–15.67)	
Lymph node status (AJCC)	N0	87.4 (69.9–104.9)	1.00	< 0.001
	N1	83.3 (67.2–98.9)	0.95 (0.47–1.92)	
	N2	28.8 (14.5–43.0)	2.02 (1.12–3.67)	
	N3a	14.2 (11.7–16.6)	3.45 (2.15–5.52)	
	N3b	7.9 (5.4–10.5)	6.68 (4.24–10.52)	
Distant metastases (AJCC)	No	29.7 (13.9–45.5)	1.00	< 0.001
	Yes	6.7 (4.8–8.5)	4.86 (3.39–6.96)	
Curative resection	Yes	54.5 (38.5–70.6)	1.00	< 0.001
	No	8.7 (6.6–10.7)	4.09 (2.99–5.59)	
Splenectomy	No	27.9 (18.1–37.7)	1.00	0.003
	Yes	11.7 (8.5–14.9)	1.57 (1.17–2.12)	
Lymphadenectomy (JGCA)	D1	20.8 (11.2–30.4)	1.00	0.836
	D2	17.9 (5.8–30.1)	0.81 (0.35–1.87)	
	D2+	17.7 (8.7–26.7)	0.79 (0.33–1.96)	
Need for blood transfusion	No	25.9 (15.1–36.8)	1.00	0.237
	Yes	13.9 (11.1–16.7)	1.12 (0.94–2.36)	
Perioperative chemotherapy	No	18.2 (6.6–29.8)	1.00	0.116
	Yes	15.6 (10.6–20.6)	1.27 (0.94–1.73)	
CTC/DTC staining	Negative	17.9 (10.5–25.4)	1.00	0.001
	CK^+^CD44^−^	22.3 (10.1–34.6)	1.00 (0.73–1.38)	
	CK^+^CD44^+^	6.7 (5.5–7.8)	2.35 (1.46–3.77)	

*Log-rank test

*ECOG* Eastern Cooperative Oncology Group, *AJCC* American Joint Committee on Cancer 2010, *JGCA* Japanese Gastric Cancer Association, *CTC* circulating tumor cells in blood, *DTC* disseminated tumor cells in bone marrow

**Table 5:** Multivariate analysis using the Cox proportional hazards model

	Category	Hazard ratio (95% CI)	*P*
Depth of infiltration (AJCC)	T1	1	
	T2	0.89 (0.39–2.01)	0.783
	T3	1.91 (1.05–4.01)	0.044
	T4	2.44 (1.06–5.61)	0.037
Lymph nodes (AJCC)	N0	1	
	N1	0.89 (0.42–1.89)	0.756
	N2	1.51 (0.78–2.93)	0.218
	N3a	2.01 (1.14–3.53)	0.016
	N3b	2.78 (1.56–4.97)	0.001
Distant metastases	No	1	
	Yes	1.95 (1.21–3.15)	0.006
Radical resection	Yes	1	
	No	1.68 (1.03–2.72)	0.036
CTC/DTC staining	Negative	1	
	CK^+^CD44^−^	0.91 (0.67–1.25)	0.553
	CK^+^CD44^+^	2.38 (1.28–4.41)	0.006

*AJCC* American Joint Committee on Cancer 2010, *CTC* circulating tumor cells in blood, *DTC* disseminated tumor cells in bone marrow

## Discussion

Liquid biopsies to detect circulating and disseminated tumor cells offer a unique opportunity to look for potential biomarkers associated with disease progression. Using a well-defined population of patients with resected gastric cancer and prospectively observed for at least 6 years we have specifically demonstrated that the phenotype of cancer cells identified in blood and bone marrow was one of the major independent prognostic factors for long-term survival.

Several previous meta-analyses suggested that CTC and DTC may have some prognostic importance. One of the more recent publications, summarizing data from 26 studies involving 2566 patients, demonstrated that such tumor cells play an important role also for gastric cancer [7]. Huang et al. showed that the detection of CTC/DTC significantly shortened disease-free survival (HR 3.42, 95% CI 2.39–4.91), while overall survival was hampered only by the presence of CTC (HR 2.13, 95% CI 1.13–4.03). Although their results clearly implied impaired prognosis due to the presence of cancer cells either in the blood or bone marrow, there was a marked heterogeneity among studies potentially attributable to the functional heterogeneity of these cells [8].

CTCs/DTCs are an important element of the ‘seed and soil’ theory explaining the concept of distant metastases associated with human malignancies [19]. However, it is clear that the functional status of these cells, corresponding to the presence of some surface markers, has a major impact on the ability to engraft into the preconditioned target niche and develop metastatic lesions. Several molecular targets were previously used to detect CTCs/DTCs in patients with gastric cancer, including CEA [20], mucin 1 [21], c-Met [22], human telomerase reverse transcriptase (hTERT) [23], epithelial cell adhesion molecule (EpCAM) [24], survivin [25], and matrix metalloproteinase-1 (MMP-1) [26]. Nevertheless, the ‘gold standard’ of cell surface markers for the detection of cancer cells was attributed to cytokeratins (principally CK8, CK18 and CK19), playing an important role in the cytoskeleton of epithelial tissues [27]. Our method combining ‘concentration’ of tumor cells by separating CD45^−^ cells (leukocyte common antigen), IHC detection of cytokeratins, and morphological evaluation under a light microscope, showed high specificity and the ability to detect 1 tumor cell per 10^6^–10^7^ leukocytes [17]. However, the process of epithelial-to-mesenchymal transition (EMT) required for primary cancer cells to acquire new features essential for the invasion of distant sites may be associated with loss of typical epithelial markers [8, 28]. Thus, cells expressing non-CK markers may potentially be more clinically relevant.

The lymphocyte homing receptor CD44 was proposed as a marker for cancer stem cells of many solid malignancies, including gastric cancer [29]. In the latter case, CD44^+^ tumors were associated with an increased risk of death (HR 1.87, 95% CI 1.55–2.26) as recently demonstrated in a meta-analysis of 4729 patients [30]. This could be at least partially attributed to the highly malignant potential of the subpopulation of CD44^+^ gastric cancer cells, as demonstrated by patient-derived tumor xenograft models [31, 32]. However, the presence of the homing receptor is relevant also for the potential tumorigenicity of CTCs. Two independent studies demonstrated that CD44^+^ CTCs isolated from peripheral blood of gastric cancer patients were able to induce tumors when injected into immunodeficient mice [33, 34]. Moreover, the majority of tumors induced in such an experimental model showed high expression levels of CD44 [35]. Finally, a small study recruiting 45 patients suggested that CTCs positive for CD44 were associated with shorter disease-free survival compared to CTCs expressing cytokeratin 19 [36].

In the current study, a well-defined population of Western patients with gastric cancer combined with long-term follow-up provides a unique opportunity to study the importance of CTCs/DTCs. A two-step procedure, combining sorting out all leukocytes (CD45^+^) and searching for cells positive for common epitopes of cytokeratins (CK8, CK18 and CK19) and CD44, demonstrated that it was not the presence of cancer cells, but their phenotype that determined patients’ prognosis. Cells positive for CD44, found either in the peripheral blood or bone marrow, were associated with a twofold higher risk for distant metastases and mortality. In contrast, cytokeratin-positive cells had no major impact on clinical outcomes. To our knowledge this is the first large-scale study that provided clinically relevant data to confirm previous experimental observations on the role of CD44 for CTCs/DTCs in patients with gastric cancer.

Our results provide an important framework for further studies focused on the role of various populations of CTC/DTC for clinical settings. It seems unlikely that detection of such cells would be used as an early diagnostic biomarker due to the low prevalence of tumor cells in either peripheral blood or bone marrow. Hence, it is much more reasonable to expect that CTC/DTC could be used to monitor response to systemic chemotherapy [37] or identify patients who would have more advantage from adjuvant treatment after surgery of primary cancer [38]. However, some important technical limitations should also be considered. Currently, there are several technologies dedicated to detection of CTC/DTC and no definite diagnostic standard has been established so far, even though immunocytochemical staining with monoclonal antibodies against cytokeratins is commonly accepted as an adequate analytical method [27, 39]. Application of A45-B/B3 antibodies that recognize common epitopes of cytokeratins increases the possibility of detecting tumor cells among the CD45^−^ population of blood and bone marrow cells. However, loss of the epithelial markers during the epithelial-to-mesenchymal transition poses the risk that some tumor cells could be undetected, thus falsely classifying patients as free from CTCs/DTCs. Therefore, further studies are urgently needed to account for the heterogeneity in the immunohistologic profile of circulating and disseminated tumor cells.

In conclusion, the results of this large prospective study recruiting Western patients with gastric cancer demonstrated that the prognostic relevance of circulating tumor cells in blood and disseminated cells in bone marrow is determined by their phenotype. Detection of cytokeratin-positive cells in either body compartment does not predict poorer prognosis.

## Electronic supplementary material

Below is the link to the electronic supplementary material.

Supplementary material 1 (DOCX 17 KB)
