# Supplementary material for: CD44+ cytokeratin-positive tumor cells in blood and bone marrow are associated with poor prognosis of patients with gastric cancer
Source: Gastric Cancer. 2018 Jul 28;22(2):264–72. doi: 10.1007/s10120-018-0858-2 (PMC6394724; doi:10.1007/s10120-018-0858-2)
Supplement: Supplementary file 1 — Supplementary material 1 (DOCX 17 KB) [file 10120_2018_858_MOESM1_ESM.docx]

| **Supplementary Table 1.**  Correlations of clinicopathological parameters with circulating tumour cells (CTC) | | | | | | | |
| --- | --- | --- | --- | --- | --- | --- | --- |
| **Parameter** | **CK/CD44 staining** | | | ***P*** | | |  |
|  | Negative  (n=197) | CK^+^CD44^–^  (n=22) | CK^+^CD44^+^  (n=9) | Negative *vs* CK^+^CD44^–^ | Negative *vs* CK^+^CD44^+^ | CK^+^CD44^–^ *vs* CK^+^CD44^+^ |  |
| Age, yrs (median, IQR) | 64 (54–70) | 61 (55–69) | 63 (59–69) | 0.349^†^ | 0.743^†^ | 0.348^†^ |  |
| Female (n, %) | 66 (33) | 9 (41) | 2 (22) | 0.397^*^ | 0.537^*^ | 0.323^*^ |  |
| Tumour grade (n, %)  well or moderate  poor | 108 (55)  89 (45) | 9 (41)  13 (59) | 4 (46)  5 (56) | 0.214^*^ | 0.541^*^ | 0.856^*^ |  |
| Primary tumour (n, %)  T1–T2  T3–T4 | 56 (28)  141 (72) | 5 (23)  17 (77) | 2 (22)  7 (78) | 0.572^*^ | 0.685^*^ | 0.975^*^ |  |
| Metastatic lymph nodes (n, %) | 148 (75) | 18 (82) | 7 (78) | 0.487^*^ | 0.857^*^ | 0.796^*^ |  |
| Distant metastases (n, %) | 42 (21) | 5 (23) | 4 (44) | 0.878^*^ | 0.103^*^ | 0.226^*^ |  |

| **Supplementary Table 2.**  Correlations of clinicopathological parameters with disseminated tumour cells (DTS) | | | | | | | |
| --- | --- | --- | --- | --- | --- | --- | --- |
| **Parameter** | **CK/CD44 staining** | | | ***P*** | | |  |
|  | Negative  (n=122) | CK^+^CD44^–^  (n=91) | CK^+^CD44^+^  (n=15) | Negative *vs* CK^+^CD44^–^ | Negative *vs* CK^+^CD44^+^ | CK^+^CD44^–^ *vs* CK^+^CD44^+^ |  |
| Age, yrs (median, IQR) | 65 (55–70) | 62 (53–70) | 61 (44–69) | 0.387^†^ | 0.652^†^ | 0.764^†^ |  |
| Female (n, %) | 44 (36) | 29 (32) | 4 (27) | 0.523^*^ | 0.471^*^ | 0.687^*^ |  |
| Tumour grade (n, %)  well or moderate  poor | 57 (47)  65 (53) | 46 (51)  45 (49) | 4 (27)  11 (73) | 0.580^*^ | 0.140^*^ | 0.086^*^ |  |
| Primary tumour (n, %)  T1–T2  T3–T4 | 35 (27)  87 (73) | 27 (30)  64 (70) | 1 (7)  14 (93) | 0.876^*^ | 0.067^*^ | 0.061^*^ |  |
| Metastatic lymph nodes (n, %) | 91 (75) | 68 (75) | 14 (93) | 0.982^*^ | 0.105^*^ | 0.111^*^ |  |
| Distant metastases (n, %) | 25 (20) | 18 (20) | 8 (53) | 0.898^*^ | 0.005^*^ | 0.005^*^ |  |

**Supplementary Table 3**

Multivariate analysis using the Cox proportional hazards model in patients subject to curative resections (R0).

|  | | | |
| --- | --- | --- | --- |
|  | **Category** | **Hazard Ratio (95%CI)** | ***P*** |
| Depth of infiltration (AJCC) | T1 | 1 |  |
|  | T2 | 0.89 (0.29-2.73) | 0.848 |
|  | T3 | 1.98 (1.09-5.67) | 0.034 |
|  | T4 | 3.67 (1.19-13.90) | 0.004 |
| Lymph nodes (AJCC) | N0 | 1 |  |
|  | N1 | 0.51 (0.18-1.43) | 0.202 |
|  | N2 | 1.63 (0.67-3.99) | 0.285 |
|  | N3a | 2.61 (1.19-5.73) | 0.017 |
|  | N3b | 3.63 (1.56-8.49) | 0.003 |
| CTC/DTC staining | negative | 1 |  |
|  | CK^+^CD44^–^ | 0.60 (0.35-1.05) | 0.328 |
|  | CK^+^CD44^+^ | 1.72 (1.08-5.10) | 0.006 |
